# Exploring the Potential of Implementing Managed Alcohol Programmes to Reduce Risk of COVID-19 Infection and Transmission, and Wider Harms, for People Experiencing Alcohol Dependency and Homelessness in Scotland

**DOI:** 10.3390/ijerph182312523

**Published:** 2021-11-28

**Authors:** Tessa Parkes, Hannah Carver, Wendy Masterton, Hazel Booth, Lee Ball, Helen Murdoch, Danilo Falzon, Bernie M. Pauly, Catriona Matheson

**Affiliations:** 1 Salvation Army Centre for Addictions Services and Research, Faculty of Social Sciences, University of Stirling, Stirling FK9 4LA, UK; hannah.carver@stir.ac.uk (H.C.); wendy.masterton@stir.ac.uk (W.M.); h.l.booth@stir.ac.uk (H.B.); d.c.falzon@stir.ac.uk (D.F.); 2 Homelessness Services Unit, The Salvation Army, London SE1 6BN, UK; lee.ball@salvationarmy.org.uk (L.B.); helen.murdoch@salvationarmy.org.uk (H.M.); 3 Canadian Institute for Substance Use Research, University of Victoria, Victoria, BC V8P 5C2, Canada; bpauly@uvic.ca; 4 Faculty of Social Sciences, University of Stirling, Stirling FK9 4LA, UK; catriona.matheson@stir.ac.uk

**Keywords:** alcohol, alcohol use disorders, homelessness, harm reduction, managed alcohol programmes, COVID-19

## Abstract

People who experience homelessness and alcohol dependency are more vulnerable than the general population to risks/harms relating to COVID-19. This mixed methods study explored stakeholder perspectives concerning the impact of COVID-19 and the potential utility of introducing managed alcohol programmes (MAPs) in Scotland as part of a wider health/social care response for this group. Data sources included: 12 case record reviews; 40 semi-structured qualitative interviews; and meeting notes from a practitioner-researcher group exploring implementation of MAPs within a third sector/not-for-profit organisation. A series of paintings were curated as a novel part of the research process to support knowledge translation. The case note review highlighted the complexity of health problems experienced, in addition to alcohol dependency, including polysubstance use, challenges related to alcohol access/use during lockdown, and complying with stay-at-home rules. Qualitative analysis generated five subthemes under the theme of ‘MAPs as a response to COVID-19′: changes to alcohol supply/use including polysubstance use; COVID-19-related changes to substance use/homelessness services; negative changes to services for people with alcohol problems; the potential for MAPs in the context of COVID-19; and fears and concerns about providing MAPs as a COVID-19 response. We conclude that MAPs have the potential to reduce a range of harms for this group, including COVID-19-related harms.

## 1. Introduction

### 1.1. Alcohol Dependency and People Who Experience Homelessness

Harmful or high-risk alcohol use is defined as alcohol consumption that is likely to lead to health problems such as psychological or physical illness due to the way that it is consumed [1]. Alcohol dependence, or the term alcohol use disorders (AUDs), are characterised by craving, tolerance, and continued alcohol consumption, despite potentially harmful consequences [1]. The harms associated with AUDs are varied: acute harms include injuries and poisoning, and chronic harms include illnesses such as heart disease, liver disease, some cancers, and stroke [2]. Social harms include issues related to housing, finances, employment, and relationships, that have occurred as a direct result of alcohol consumption [3,4]. The World Health Organisation (WHO) estimates that AUDs affect approximately 237 million adult men and 46 million adult women globally [5]. Approximately three million deaths occurred as a result of harmful alcohol use in 2016 [5]. In 2019, there were 7565 alcohol-specific deaths in the UK [6]. Across Scotland, 1190 people died of alcohol-specific causes in 2020, an increase of 17% from 2019 [7].

AUDs are not seen equally across all social groups, with people who experience homelessness being more vulnerable to AUD and associated harms (such as seizures; withdrawal; chronic liver disease; premature death; injuries and poisoning; and social exclusion and discrimination [3,8,9]. Statistics on the prevalence of AUDs among people who experience homelessness vary, but estimates are high at 58.5% [10]. Data are limited for women. Higher prevalence rates have been shown to be linked to structural disadvantage and marginalisation related to socio-structural factors such as poverty, racism, and colonisation [11,12], and compounded by stigma and discrimination [12]. Alcohol may be implicated in the pathway to homelessness and may be used as a way of coping with current or past challenges and traumatic life circumstances that precede or follow homelessness [13,14,15]. Despite higher levels of AUDs and alcohol-related harms, people who experience homelessness access healthcare services, including mental health services, less often. When they do it is often at crisis point, attending accident and emergency instead of primary care services [16]. Treatment options for people who experience homelessness and AUDs are limited, and abstinence-focused treatment programmes can be difficult to comply with because of unrealistic conditions and/or undesirable goals [17].

Many people who experience both homelessness and AUDs express a preference for harm reduction options where they have a choice in how best to manage their alcohol use [18], for example by using safer drinking approaches rather than enforced abstinence, which can result in repeated difficult experiences of withdrawal and detoxification [12,18]. There is, however, a lack of alcohol-specific harm reduction for this group, particularly in comparison to initiatives for illicit substances [11,19,20]. This is despite recent evidence concluding that these strategies are a viable treatment option for AUDs in some circumstances [21]. Existing alcohol harm reduction typically focuses on population-level strategies such as low-risk drinking guidelines, and policies such as pricing that are intended to encourage moderation within the general population [11,19], although there is some evidence of positive effects of minimum unit pricing for people with AUDs [22]. These strategies and policies are unlikely to meet the needs of those whose drinking patterns do not fit with drinking norms, such as people who experience homelessness with AUDs [19]. This has led to calls for tailored interventions for this group, such as safer use, in order to reduce adverse effects of alcohol without requiring abstinence [11]. Research is increasing in this area, with positive results concerning alcohol management programmes and the combining of behavioural harm reduction and pharmacological approaches [23,24].

### 1.2. Alcohol Use Disorders, COVID-19, and Managed Alcohol Programmes

An additional concern for people who experience homelessness and AUDs is COVID-19 which is responsible for a global pandemic [25]. They are at significant risk of being negatively affected by COVID-19 due to increased likelihood of severe disease and/or death [26,27]. Many such individuals have multiple morbidities or compromised immune function and are therefore additionally vulnerable [28,29]. Risks specific to people with AUDs during the pandemic include: difficulties accessing alcohol as a result of shop closures/changes in opening hours; challenges with managing new pandemic rules such as the wearing of masks in shops; loss of income from street begging/pan handling to purchase alcohol; restrictions on using cash rather than credit/debit cards; sharing alcohol; a street drinking culture making social distancing and/or self-isolating difficult; and potential increased intake of non-beverage alcohol (i.e., hand sanitiser or rubbing alcohol) [12,30]. There is a risk of withdrawal if people with AUDs cannot maintain their supply, leading to serious health consequences such as seizures and even death [19,31]. Alcohol may also be substituted for illicit drugs which can increase harm and risk of death [19,32].

Additional problems have been identified for people who are homeless and use substances during the pandemic. These include the inability to shield or isolate oneself due to: not having safe housing; difficulty with social distancing; poorer access to resources to protect health (such as personal protection equipment (PPE); and potential medication shortages and/or disruption to health services such as hepatology appointments [20,33]. Breaking lockdown rules in Scotland has had the potential to incur criminal justice sanctions if repeated on a number of occasions, with the likelihood of fines being issued 12 times higher in deprived areas [34]. To address the increased risk of harm in the context of COVID-19 there has been a call for rapid changes to substance use services and treatment [35,36,37]. Global changes that have been identified relative to people with AUDs include: ensuring people with AUDs have access to COVID-19 screening and testing [38]; updated guidance about how best to provide AUD treatment while reducing the risk of virus spread in shelter/hostel settings [39]; increased access to withdrawal management medications and other medications such as disulfiram [38]; safer drinking guidance [40]; and operational guidance for provision of managed alcohol [30].

Managed alcohol programmes (MAPs) are harm reduction services that provide regular and measured doses of alcohol throughout the day and aim to improve health and social outcomes by providing shelter or housing, education, social support, and other services [12]. MAPs are designed for people who experience homelessness and AUDs, and who are not using higher-threshold addictions services (i.e., abstinence-based treatment, detoxification, and other services that require abstinence/structured treatment and appointments which discharge people for non-attendance) [12]. Currently most MAPs are based in Canada, although they exist elsewhere, for example in Ireland [41] and the United States (US) [42]. There are currently 39 MAPs across Canada [43] and, although they are most common within residential accommodation, there have also been positive outcomes reported within a non-residential programme [15]. As Ivsins et al. [11] describe, many MAPs operate ‘under the radar’, historically and still today, because of public controversy concerning them.

Studies in Canada now demonstrate positive results from MAPs, with participants experiencing lowered alcohol-related harm such as: reduced withdrawal seizures and non-beverage alcohol intake; less harmful patterns of use; improved safety and fewer accidents; improvements in relationships; and a reduction in contact with police and emergency health services [44,45,46,47,48,49]. While those involved in a MAP have daily drinking, they have less overall alcohol consumption [47]. MAPs have been described as safer environments compared to hospitals and prisons [45], and as safe places for healing, reconnection, and the realisation of individual goals [3]. While there is limited evidence regarding the impact of MAPs during the COVID-19 pandemic, evidence from the US suggests MAPs facilitated isolation/quarantine requirements, and reduced harms associated with alcohol withdrawal [42].

In the UK there are residential accommodation services that provide support for people with AUDs, and some include support with managing alcohol use [50]. While no MAPs formally existed in Scotland during this study, one is soon to open in Glasgow run by Simon Community Scotland [51]. Carver et al. [50] explored the feasibility and acceptability of implementing MAPs in Scotland and concluded that MAPs could address a gap in alcohol harm reduction nationally as part of a system of care to reduce alcohol-related harm for structurally vulnerable people. Given the lack of published evidence regarding the provision of MAPs during the COVID-19 pandemic, this study made hypothetical links between MAPs and COVID-19 to explore their potential.

### 1.3. Study Aim and Research Questions

This mixed methods study explored stakeholder perspectives concerning the impact of COVID-19 on people experiencing homelessness and AUDs and the potential utility of introducing MAPs in Scotland as part of a wider health/social care response for this group. The aim of this study was, most specifically, to explore the potential of MAPs within The Salvation Army (TSA) third sector homelessness services in Scotland. The study was conducted over a period of six months, between May and November 2020, in collaboration with TSA, which wished to examine how MAPs could be used to address the needs of its clients during the pandemic. The study had the following research questions which were addressed using both quantitative and qualitative methods:

- How is COVID-19 impacting people experiencing homelessness and AUDs in Scotland, including access to services and alcohol?
- How might the introduction of managed alcohol programmes (MAPs) in Scotland reduce COVID-19-related (including risk of infection/transmission) and wider risks/harms?

We report all the quantitative data collected and present one of the most significant themes from the qualitative dataset: MAPs as a response to COVID-19, with five associated subthemes. Further qualitative papers are in preparation to address the remaining themes of training and workforce implications, and generic implementation considerations, including non-COVID-19-related service development.

## 2. Methods

### 2.1. Study Design and Ethics

A mixed methods design was used involving case record reviews, semi-structured interviews, and meeting notes from a group set up to explore potential implementation of MAPs within TSA. Four service settings (i.e., facilities) were selected to undertake data collection capturing diversity of client and staff experience, geographical locations, and service models: two large ‘Lifehouses’ (hostels/shelters), a Housing First setting (permanent supportive housing [24]), and a city centre ‘drop-in’. Given the demands on these settings during COVID-19, the research team worked closely with TSA to identify settings where managers were supportive regarding a research study, and were able to support study recruitment, given the priority was to maintain essential services. Staff absences were also taken into account in these decisions.

A gallery manager and artist in residence were commissioned at the start of the study to create a range of visual images to represent emerging themes for knowledge exchange purposes (to more effectively communicate the findings with a wide range of audiences online and bring a strong lived experience dimension to the study). As part of the study, a strategy group (SG) was established consisting of members of the research team, TSA managers, the gallery manager and artist. The SG was responsible for overall management of the study and met 12 times. Three of the settings participated in the case record review and interviews, with quantitative and qualitative data being collected concurrently; the fourth was only involved in interviews. Ethical approval for the study was granted by University of Stirling’s General University Ethics Panel (GUEP; paper 917) and the Ethics Subgroup of the Research Coordinating Council of The Salvation Army (RCC-EAN200709). Rigorous risk assessments were conducted for face-to-face data collection, as per TSA and University of Stirling COVID-19 protocols.

### 2.2. Case Record Review: Eligibility and Measures Collected

Consistent with previous studies [50], the aim of reviewing these case records was to illustrate experiences specific to those who would be eligible for MAPs by exploring how they managed during the early COVID-19 regulations and to identify any alcohol-related health issues. Quantitative data were collected via case record reviews of a subset of individuals accessing three of the study settings (i.e., those who met the study inclusion criteria). For inclusion in the case record review, individuals had to have experienced homelessness (or be at risk of) and have alcohol as their main substance use problem: they did not have to be formally diagnosed with AUD. Prior to case record review data collection, all eligible clients in the settings were provided with a letter explaining the study and the intention to collect non-identifiable information from client case records. All clients were asked to contact a member of staff if they wished to participate (formal opt-in consent). To reduce the burden on frontline staff, a TSA manager who was part of the SG collated anonymised participant information using TSA’s ‘Atlas’ client management system and generated an Excel spreadsheet.

The records of 12 clients who met the study inclusion criteria were reviewed and collated. We aimed to collect data from as many eligible people as possible, with initial estimates of around 30. Due to the transient nature of the group, however, the high number of people for whom drug use was the main concern (see [52] for more detail on polysubstance use in the Scottish context), the challenges of collecting data during a pandemic, the formal opt-in consent, and the short study timeframe, we were only able to access the case records from 12. Data were collated on: demographics (age range, gender, housing status, income); current alcohol/other substance use; physical/mental health problems; alcohol withdrawal symptoms; AUDIT score [53]; and data relating to COVID-19 (e.g., positive COVID-19 test result; abiding by social distancing/hygiene recommendations; and following lockdown rules) and then shared with the research team. Some data were entered into the spreadsheet as they appeared in the case records: others were calculated/amended as required. Data were analysed in SPSS using descriptive statistics to provide an overview of the population, alcohol use, and COVID-19 experiences.

### 2.3. Interviews: Recruitment, Process, and Data Analysis

Qualitative data were collected via interviews with service managers, frontline staff, potential beneficiaries/clients who would meet (or would have met) the inclusion criteria for MAPs, and a range of external stakeholders (such as those working in healthcare, local councils, government, police or other third-sector/not-for-profit organisations), with a sample size of 20–45. External stakeholders were identified through the research team’s networks of those working in the field and included national organisations, statutory and third-sector/not-for-profit organisations. Semi-structured interviews were conducted by four members of the research team (HC, WM, PM, JD) between July and October 2020. Purposive sampling was used to select individuals based on gender, role, and organisation, to ensure the sample reflected a wide range of views/experiences. Service managers in the four settings were asked to provide a list of frontline staff to be contacted for an interview. Service managers did not know which staff members had actually been approached by researchers or interviewed for the study. Managers, frontline staff, and external stakeholders were contacted by email and invited to participate in the study. Client participants were identified by staff in the settings and asked if they would be interested in participating in an interview.

All potential participants were provided with an information sheet and assured that participation was voluntary. Written or verbal informed consent was granted at the beginning of each interview. Client interviews were conducted either face–to-face in the settings or by phone (JD). Staff/stakeholder interviews were conducted via phone (HC, WM, PM). All but one of the interviews were audio recorded and lasted an average of 51 min. Interview schedules differed for each group and covered, for example, views on the need/potential for MAPs in the context of COVID-19 (for full list see Supplementary file S1). After each interview, participants were provided with a debrief sheet to provide further information and support available, and client participants were provided with a £10 ‘thank you’ high street shopping voucher to acknowledge their time. Detailed field notes captured researcher experiences and reflections of the interview as a way of enhancing reflexivity [54], which supported changes to the schedule to enhance clarity, ensuring the questions were relevant and guided by initial findings.

Data were transcribed in full and analysed using Framework analysis [55], which is suited to policy-relevant research and provides a structured and transparent method of data analysis [56]. Transcripts were combined into four separate participant-type datasets and read in full, then coded line by line in NVivo 12 by four researchers, with a further researcher checking coding for consistency across each coder (the study had to be completed within a six-month period, which is why a team of researchers undertook the analysis). This collaborative process provided opportunities for discussion on anything that was unclear or could have different interpretations. The research questions guided the data analysis but data were also coded inductively to allow new ideas to be explored and added to the framework. After coding the first six transcripts, the initial thematic framework was developed (H.B., H.C.) and used to code remaining transcripts. Data were then rearranged into themes/subthemes with illustrative quotes selected. Each transcript was then re-read for completeness to ensure no themes had been missed.

### 2.4. Use of Meeting Notes

Using the same framework and process as participant interviews, all SG meeting notes were included as data in order to understand wider staff/manager concerns regarding potential implementation challenges and workforce needs. All members present in SG meetings gave written consent. The meeting notes were transcribed and anonymised by a member of the research team. These data are incorporated in the findings below.

### 2.5. Creation of Paintings

As noted above, a gallery manager and artist in residence were commissioned to create a range of paintings to represent the emerging themes of the study while data collection occurred. The series of paintings were co-curated as a novel element of the project to aid with study dissemination during a time where online meetings had replaced face-to-face gatherings. From previous work undertaken on MAPs [50], we understood the importance of capturing themes visually, as well as in writing, given the emotional context of the work (see Pauly et al. on the importance of safety and community [45], which only intensified during COVID-19). We were keen to ensure that study outputs had a visual and creative dimension to them in order to ‘bring the work to life’ for the range of audiences who are interested in MAPs. The artist and gallery manager signed confidentiality statements and no identifying images were created. They attended most of the SG meetings and listened to the discussion of the issues/concerns that were being raised through the study. This process resulted in a series of ten paintings, which we will display once COVID-19 restrictions end in order to facilitate discussions on study findings with public audiences. Three of these images are included in this paper to illustrate elements of the study’s findings.

## 3. Findings

### 3.1. Quantitative Data

The case record review highlighted the complexity of people’s lives, including: high rates of alcohol use; physical/mental health problems; polysubstance use; and experiences of COVID-19. Table 1 shows the demographic characteristics. Physical health problems were reported for almost all (*n* = 11; 92%). Mental health problems were reported for all 12, with depression and anxiety being most common (*n* = 11; 92%). Multiple mental health problems were reported for 83%.

Table 2 provides an overview of alcohol and drug use. Alcohol use was high, with most participants consuming at least 20 units per day, and half drinking every day. All participants had been drinking alcohol for at least 10 years, with 11 reporting drinking for at least 20 years (92%). AUDIT scores were reported for 11 of 12, with scores ranging from 13–36, and a mean score of 30 and a median score of 33 suggesting likelihood of alcohol dependence. Only two of the 11 reported AUDIT scores of less than 20 [53]. Daily withdrawal symptoms were reported for all 12 participants, with seven having had seizures (58%). Current drug use was reported for all 12 participants, with seven (58%) reporting using more than one substance, although it was unclear from the case notes whether this was concurrent/simultaneous use or not.

Table 3 provides COVID-19-related data. At the time of data collection, only one person had experienced COVID-19 symptoms and had been tested, and only one had been shielding. Nine broke lockdown rules in order to consume alcohol, either having friends in their home to drink, leaving their accommodation to buy alcohol, or drinking on the streets.

Overall, these data highlight the high rates of alcohol/drug use, mental/physical health problems, and COVID-19 challenges experienced in this small sample.

### 3.2. Qualitative Data

A total of 40 interviews were conducted with 19 external stakeholders, eight TSA service managers, seven TSA frontline staff, and six clients. We were unable to recruit more clients due to the lack of eligible participants in the settings, challenges of doing online/telephone interviews, and lack of staff time for recruitment. Table 4 provides interview participant characteristics.

Qualitative analysis of interviews and meeting notes generated five subthemes under the dominant theme of ‘MAPs as a response to COVID-19′: changes to alcohol supply and use and polysubstance use; COVID-19-related changes to substance use and homelessness services; negative changes to services for people with alcohol problems; the potential for a MAP in the context of COVID-19; and concerns about providing MAPs as a COVID-19 response.

#### 3.2.1. Changes to Alcohol Supply and Use and Polysubstance Use

Data captured in the meeting notes suggested that changes to use of substances were observed, with some people moving from alcohol to drug use, and vice versa. Other interviewees highlighted that, rather than alcohol use increasing, use of illicit drugs in addition to alcohol was the main issue of concern. This was supported by the case record data where all clients had used illicit drugs, in comparison to previous similar research where 58% of clients reported illicit drug use [50]. One stakeholder participant spoke about the early weeks and months of the pandemic in the following way:

*What we have got now is a population concentrated in the city centre… within a number of hotels where we’ve got a mixture of folk whose primary substance use is now being tested. Those where alcohol might have been a fundamental substance have now suddenly been introduced much more readily to street valium, etizolam, much more readily available heroin and cocaine, and vice versa*.

(Stakeholder 15)

A client participant also commented on polysubstance use:

*What you’ve got to understand is it’s not just [alcohol] that they are taking, they are taking drugs as well… They were taking drugs and drink before the pandemic*.

(Client 5)

Another participant talked about the challenges experienced by clients in accessing alcohol during the pandemic:

*I would say that, certainly, the lockdown posed a lot of kind of issues initially in terms of being able to access alcohol. A lot of the people that we work with rely on begging and stuff to get money for their alcohol, for their daily alcohol use, and that has been an issue because of COVID. People don’t carry the same amount of cash, like coins and stuff around and then obviously lockdown was a huge issue… because of lockdown restrictions*.

(Manager 5)

Other participants said there was no significant change in alcohol or drug use stating: *“The drug market never really changed”* (Manager 4), and *“Alcohol consumption on the whole stayed fairly static”* (Manager 1). A member of staff working in the NHS (Stakeholder 14) stated that: *“There is no real evidence of increases in use at the moment”,* but added that they believed there would be in the future. Some clients reported that they had taken the opportunity of lockdown to reduce their alcohol use, with one client saying COVID *“got the ball rolling”* (Client 1), something two managers echoed:

*There was a group of people who became very aware that this was a really unique point in time and what we actually saw was a lot of people taking really good care of themselves and reducing their alcohol consumption.*

(Manager 1)

*When the money suddenly becomes more difficult, or the place to drink becomes more challenging to achieve, is it worth all the effort? It’s that effort and benefits of living, making those choices, compared to deciding to change. COVID has made living that lifestyle a bit more challenging and, maybe for some people, opened up the door to let’s look and see if there is a different way of going about day-to-day existence.*

(Manager 6)

#### 3.2.2. COVID-19-Related Changes to Substance Use and Homelessness Services

The pandemic inevitably changed the way that substance use and homelessness services supported clients, since indoor, one-to-one support was often no longer feasible. The necessity of service adaption was instantaneous and, although challenging, many services did so in creative ways. One of the major changes during the pandemic was the way that services relaxed strict rules on abstinence. Many homelessness organisations do not allow clients to drink alcohol onsite, and changing this rule was described by participants as a *“massive, massive shift in organisational structure”* (Manager 4), and a *“curve ball”* (Stakeholder 16), because it *“flew in the face of a lot of how (staff) felt that treatment should be done”* (Manager 1). This change was deemed necessary because, if clients were not allowed to drink in their accommodation, but also not allowed to go offsite, this represented a *“catch twenty-two”* (Stakeholder 13). One of the reasons this shift happened was to mitigate the risk of alcohol withdrawal. According to one participant, there had to be *“fail-safes in place if you knew someone was going through alcohol withdrawal”* (Manager 7). One participant explained their approach to one client: *“This is not about keeping them drunk or intoxicated, this is about keeping him alive”* (Stakeholder 10). Another stated: *“…this is to save a life… it’s the same with providing somebody with naloxone.”* (Manager 4).

A number of staff and stakeholder participants commented on the move online, which worked *“really effectively”* for some individuals to increase reach/accessibility, with recognition too, however, that such an approach was not suitable for all clients, nor all service or support types. One stakeholder stated that many people had been *“quite receptive”* to such changes, with a client participant stating:

*It’s actually quite good compared to the past because in the past, like, I never really used to engage with any of the services.*

(Client 1)

Other participants spoke about how people who had been rough sleeping were moved into hotels and bed-and-breakfast accommodation. Indeed, flexible and solution-focused approaches to getting people into accommodation were *“sped up”*:

*It’s a case of, “Right so this is what we need, so how do we make that work?” Whereas previously that level of flexibility wouldn’t have been there. It would have been, “No you need to go through a, b, c, before we can discuss c.” Things can happen quicker, people are more likely to listen to things that previously you would be, like, no chance will that ever happen. Now there are people who feel a bit more liberated. Innovation is encouraged*.

(Manager 7)

Some harm reduction services (with a drugs focus) were modified as the onset of COVID-19 required swift service adaptation:

*That assertive outreach format has worked quite well. It was obviously something that was already in train anyway. But, certainly, the pandemic has sped up delivery*.

(Stakeholder 17)

**Figure 1:**
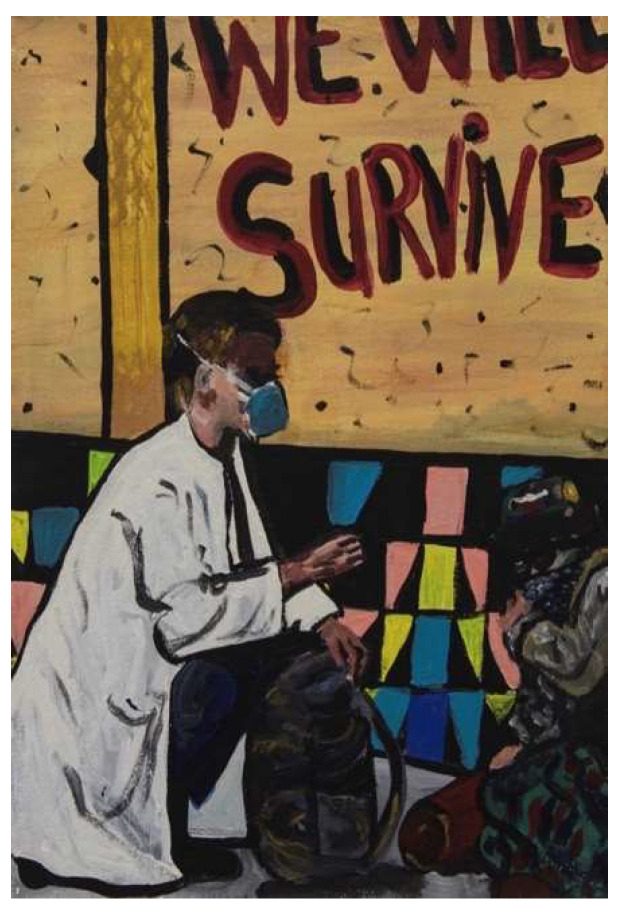
Painting 1 showing services changing to being provided on the streets.

The painting below (Figure 1) depicts the COVID-19-related changes to substance use/homelessness services and, in particular, the increase in outreach services.

The increased partnership working between the third/not-for-profit sector and statutory health and social services was deemed necessary during the pandemic (as highlighted elsewhere [52]) because significant gaps in provision needed to be closed. There was a mutual understanding that reducing risks to clients was essential. Two participants explained how increased communication between organisations, and access to technology, were central to facilitating these successful partnerships:

*A big lesson from the response to COVID has been developing much stronger partnership ties and a realisation that we can deliver a lot more, and a lot more effectively, when we are working together. We can reach folk that previously we’ve struggled to offer an effective service to*.

(Stakeholder 12)

*The partnership working has really delivered, and that is principally because we’ve been all aligned towards a singular objective. There has been a great deal of investment, effort, and willingness to work as collaboratively as we can to streamline things. There has been a lot of elements that have kind of enhanced the way in which we collaborate, such as having access to each other through use of technology. It’s meant we can very quickly have conversations. A lot of the challenges from the past have been stripped away in terms of information sharing.*

(Stakeholder 17)

Further, shifts in power dynamics between clients and staff were described, stemming from people looking out for one another:

*What also happened was that service users in a building were as responsible for taking care of the staff as the staff were at taking care of them. So, it’s not just this feeling that support works in one way here. Like, all of a sudden, I have to make sure that I am looking after you as much as I can to keep you safe. And it was probably the time when there was a bit more equity than there has ever been within services. That equity led people to make much more compassionate, much more insightful, much more reflective interventions with each other, in every direction. You saw a lot of people reduce use, reduce recklessness because, all of a sudden, you need me.*

(Manager 1)

#### 3.2.3. Negative Changes to Services for People with Alcohol Problems

Although there were some changes to services that were deemed positive by participants, the pandemic also highlighted limitations, particularly for alcohol services which were described as catering for *“second class citizens in the world of the health service”* (Stakeholder 4). Although interviewees reflected that gaps in alcohol services existed pre-COVID-19, the pandemic was said to have *“shone a light”* on the issue:

*What we found was that, at the start of COVID, alcohol services were an early casualty of COVID. We had many reports of alcohol services diminishing very quickly, and there were examples of alcohol facilities being used for other purposes. I think there were examples of responses to other drugs prioritised ahead of alcohol*.

(Stakeholder 3)

Participants said that the focus on drug services was understandable because of the rising drug deaths, but they believed that this was often viewed as a *“bigger issue”* in Scotland:

*A lot of worry, there is a lot of concern… more so around drug deaths. Why there is not the same level or worry and concern around alcohol-related deaths I don’t know.*

(Stakeholder 5)

Some of the clients interviewed stated that this gap in services had made it hard for them to get support: *“I’m going to have to wait months and months and months down the line, before anything gets done, so it’s just a rollercoaster*” (Client 1). Others were advised to continue drinking due to lack of support available:

*The advice that they were given, by and large, was continue on alcohol. It’s not safe to currently detox you. We can’t put the support in around you. You are going to be socially isolated, this isn’t a good time to try to detox.*

(Stakeholder 13)

Some clients expressed that the changes to move to online provision had limited their support. One staff member spoke in depth about trying to support a client to join recovery meetings online but, ultimately, he *“didn’t feel comfortable attending them so he just stopped”* (Staff member 3). As well as reduced access to alcohol support, clients experienced increased social isolation:

*I’ve not even got a bus pass, I’ve not got this, I’ve not got that, I can’t get fuck all. Once I’m locked down that’s me. I’ve got no money or nothing […] I’ve not really had as much help as what I would like*.

(Client 2)

**Figure 2:**
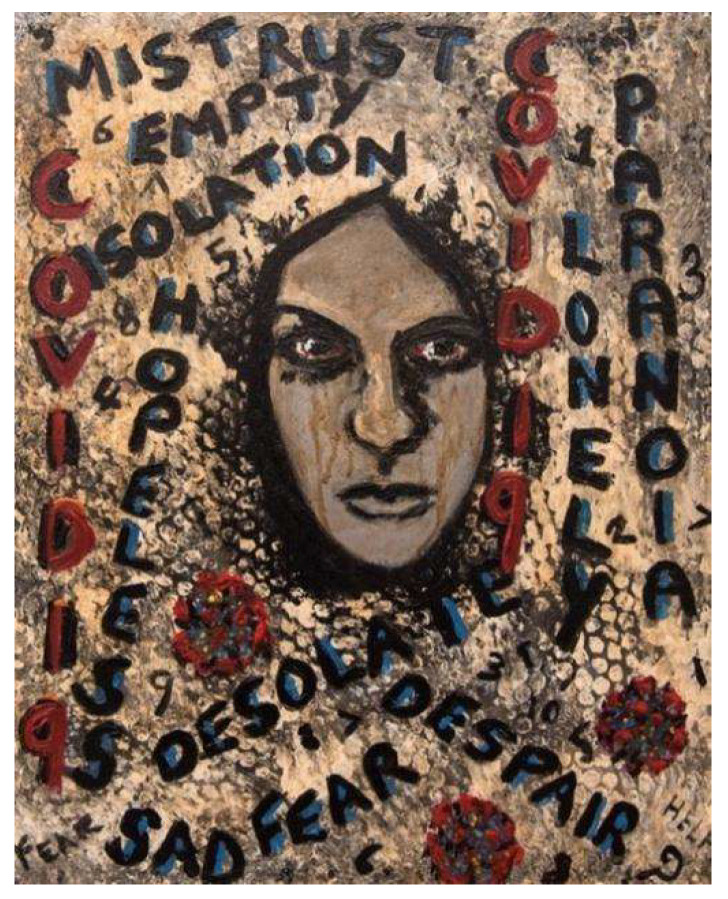
Painting 2 illustrating emotions experienced by people during the pandemic.

The painting below (Figure 2) was created to show a range of feelings and emotions experienced by clients of substance use and homelessness services during the pandemic.

Stakeholder participants reported their perception that individuals were not coming in for treatment in the same way, or were presenting with more serious symptoms at a later point in time:

*When lockdown first happened we noticed a massive drop-off in referrals and the implication was that patients weren’t coming, not because there was unlikely to be a problem, but that they were suffering somewhere else.*

(Stakeholder 11)

*We saw a significant reduction probably in the number of people presenting with liver problems initially but, again, after a slightly longer time, maybe after about six to eight weeks, we’ve now seen these folks coming in and possibly more unwell than they would have been otherwise in that they have been staying at home or were staying at home and not presenting to medical services with advanced liver disease. They are actually coming to us sicker than they maybe would have done prior to the pandemic.*

(Stakeholder 9)

#### 3.2.4. Potential for a MAP in the Context of COVID-19

Participants discussed feasibility of MAPs in Scotland, in terms of different service models (residential/drop in), the need for clear guidelines/rules and clinical input, funding of alcohol and eligibility. These data will be presented in a separate paper due to their detail and relevance for those implementing MAPs. In this section we present data specific to COVID-19. Participants discussed that MAPs could be important in the context of COVID-19 to reduce the risk of disease transmission, while also acknowledging that the rate of infection had not been as severe as first feared among people experiencing homelessness in Scotland. One manager commented that some clients did not change:

*(Clients) weren’t really working with COVID guidelines, centre guidelines, the government guidelines. They continued to live their life the way they would normally live their life. And a few did actually self-isolate but that was probably those clients who are not actively in addiction. The other clients who were maybe taking a drink of alcohol, or using substances, they just continued.*

(Manager 8)

There was an overall consensus among participants that MAPs could mitigate risk of infection:

*In terms of looking at infection control, it’s enabling people to keep themselves safe but also to prevent their need for running around to access alcohol, or funds for alcohol, which would put them at risk in terms of (being) unable to actually maintain social distancing. A MAP in that context has a double benefit, both in terms of the individual, reduced risk of COVID and alcohol-related harm, but also a public health benefit to others in less likely onward transmission of any infection.*

(Stakeholder 12)

*I reckon it would reduce it vastly, aye, because you are mixing with people, you are looking for opportunities to raise some money there or maybe a bottle will be on the table and then you are drinking out of the same bottle.*

(Client 3)

One manager highlighted the challenge that many clients experienced in complying with government COVID-19 restrictions, something supported by our quantitative data where nine clients reported breaking rules. In the case records, one client reported that they did not isolate when they were required to. Indeed, staff highlighted that, as well as mitigating transmission, a MAP could also address issues with accessing alcohol while requiring to self-isolate, given how difficult this would be for someone with an AUD:

*The thing with the MAP is, if it was on offer, it could stop somebody having to leave a building. The person might not necessarily put reduced risk of infection at the top of their list. But staff might. The priorities of the individual might be different.*

(Manager 1)

*A lot of the guys that I work with, you know, are street beggars, and a vast majority of their time was spent out on the streets trying to create money, so it was really, really difficult for them. Every time they were going out they were getting either fined or they were getting ushered back into the service.*

(Manager 4)

MAPs provide other support and promote engagement with other elements of services, which could also be beneficial during COVID-19. One participant explained that a MAP could:

*…get people the proper support, get them engaged without having to hide their alcohol, having to lie about their alcohol intake. A MAP takes all that away. It reduces the stigma. And I believe that relationships then can be built to, you know, access other support.*

(Manager 4)

**Figure 3:**
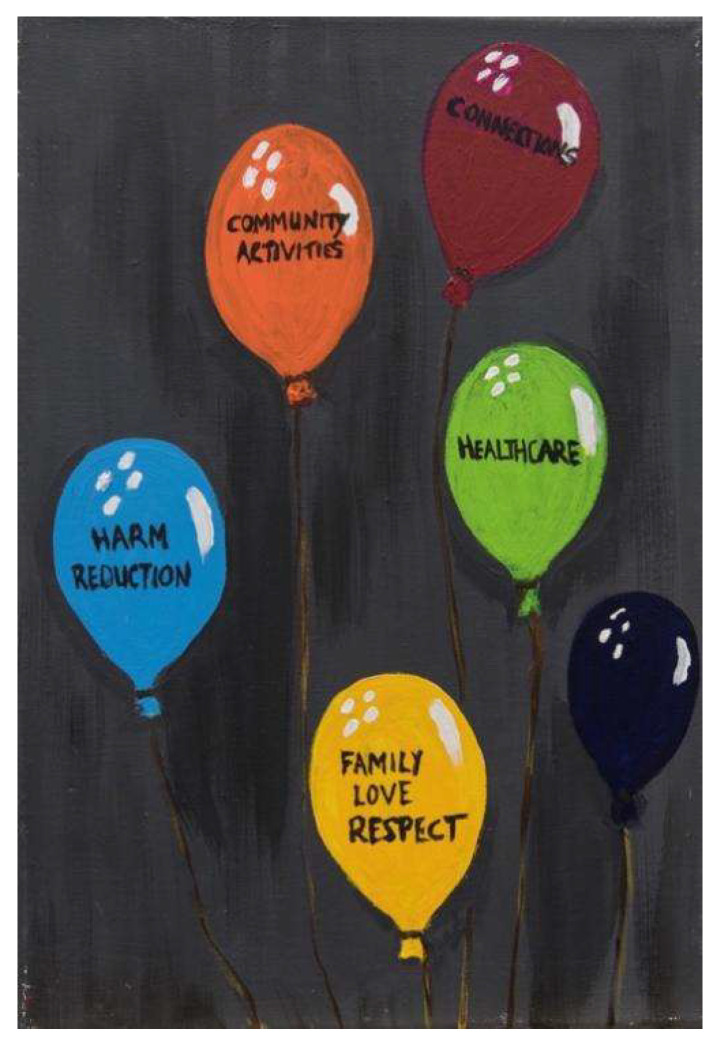
Painting 3 showing features of MAPs.

One external stakeholder spoke about how MAPs could reduce isolation concerns and provide *“meaningful relationships, interactions, activities going on, compensating for what we take away”* (Stakeholder 18), referring to the forced closure of recovery cafes and communities due to the pandemic. The painting below (Figure 3) illustrates that MAPs are not solely about providing alcohol, but also include reducing isolation and barriers to healthcare, and treating people with respect and dignity.

With growing awareness of the need for better harm reduction relative to alcohol, some participants spoke about how the pandemic presented an opportunity for MAPs to be implemented as part of national and city-wide responses. This was highlighted as something that would differ markedly from what existed pre-COVID, where alcohol services were described as *“quite linear, quite regimented”* (Manager 7). Some participants spoke about how the fear of COVID-19 had *“brought a sense of urgency to the table that wasn’t there before. If we think it will save lives, we are going to give it a go.”* (Stakeholder 5)

#### 3.2.5. Fears and Concerns about Providing MAPs as a COVID-19 Response

As well as opportunities presented by COVID-19 in relation to MAPs, participants also spoke about their uncertainties, fears or concerns. Conversely to comments at the start of this findings section, some believed that there would be *“a lot more hoops”* (Stakeholder 1) in terms of regulatory barriers to implementation. There were a number of fears raised in interview data which are illustrated by this participant:

*People are going to be frightened of how safe the client is. How safe they are in that kind of relationship. What their responsibility is. People in this moment in time, and we are talking about a MAP within this framework as an intervention around COVID, people feel really, really unsafe at the moment. What you have is a lot of community… I don’t want to use the word trauma because it’s flippantly used. But there are a lot of people in communities feeling isolated, unsafe, and scared. And we are just starting to come out of that feeling now. The biggest barriers are going to be that feeling of safety. That basically people’s sense of vigilance is going to be really heightened. The idea that you are giving the thing that you know is causing harm to the person, to NOT cause them harm, seems nonsensical.*

(Manager 1)

Other concerns involved questions about who would fund a MAP, with *“conflict”* predicted if staff members needed to buy clients alcohol. One staff member believed that there would need to be a lot of evidence to ‘back up’ the programme with external funding in place. Another participant discussed the challenges of identifying funding, stating that it *“straddled different funding streams and different strategic priorities*”, falling between homelessness and addiction services (Stakeholder 13). Although reducing risk of transmission was spoken about as a potential benefit of MAPs, conversely participants described the challenges of enforcing social distancing, and questioned whether this was feasible within a MAP:

*It’s kind of counterintuitive. To pool people together in one place increases the risk of transmission in that, you know, just like in care homes… having a lot of old people together. If you pool a lot of homeless drinkers together in one place, the risks of putting them into a hotel say… what risks does that bring? Do people who have a drink problem continue to drink? Do they respect social distancing, particularly when they are intoxicated?*

(Stakeholder 4)

Client and staff ‘buy-in’ was another issue raised by a number of participants. Some said that clients would react positively to the idea of a MAP, another stated that it could be hard to convince clients of the worth of a MAP given that it might disrupt their day-to-day lives:

*Most of our clients, that is part of their lifestyle. They’ve been begging for twenty, thirty years. They are used to drinking in big groups… I don’t know how we could sell it to them because none of our clients are sort of COVID aware. No matter what we say to them about, you know, “I’m going out for a walk”, “Do you really need to go to the shop?” And you know they don’t like being restricted or being told that they shouldn’t be going out.*

(Stakeholder 10)

It was also evident that staff training would be necessary to help secure buy-in among staff members involved. In SG meetings. challenges were raised relating to a lack of staff knowledge about harm reduction approaches to alcohol and MAPs. In particular there were concerns relating to the move away from abstinence-based approaches, and the need for training to be provided for staff on the reasons why harm reduction approaches were suitable. This was also illustrated in interviews with managers and staff members:

*Because of COVID, we changed our policy about buying alcohol. (…) That actually caused a bit of a divide between the staff team. There is something about people’s understanding of it being a harm reduction approach and not just about going out and buying someone a carry-out, do you know? That was the difficulty. There is also that thing about individual people’s values base and I think it’s really important that we do the webinar and training so that people are clear on the reasons why. And being able to look at the evidence where it’s worked in other areas as well, because you want to bring people alongside you, rather than bringing in interventions when people don’t really understand the need for them, what they are and what they are not.”*

(Manager 5)

*We spent a training day looking into the model and how it would best help our service users and absolutely I think they would buy into, you know, delivering the model and people would go and get trained up and take an interest in it as well.*

(Staff member 1)

Given that MAPs run in communities, participants also spoke about the need for community buy-in (meaning local neighbourhoods, residents, businesses), to *“bring people alongside you”* (Manager 5), which some stated might be *“tricky”* until services become more commonplace:

*This is a really critical element to the success or otherwise… getting buy-in from the local community. And my starting point with pretty much everything is that if people understand what you are trying to achieve, and they can see the logic, then it doesn’t take very much to be bought in. It’s where something lands on their doorstep, they don’t understand it, they see the fallout from it, and they feel as if it’s something that has been forced upon them that they kind of… it detrimentally impacts on their day-to-day lives. It is about engaging that community potentially and, again, it’s challenging with the COVID restrictions. You might not be able to have a town hall event but you’d be able to do things through the virtual medium to allow people to hear what you are doing, how you are doing it, what the evidence is to show that it’s effective, what the results have been from elsewhere*.

(Stakeholder 17)

Finally, while many participants were of the opinion that MAPs could be successful during COVID-19, they stressed that MAPs should not be put in place purely as a response to the pandemic. In their view, MAPs should be created as longer-term solutions, and not removed post-pandemic with return to abstinence-based models. These quotes illustrate the view that service provision pre-pandemic did not meet the needs of this client group, so MAPs can address this gap by providing important options and alternatives:

*COVID-19 brings a couple of aspects of a MAP into focus, you know? The vulnerability to infection and risk of spreading infection by some individuals, but in fact it’s a broader need. It was a need for something to address the needs of these individuals prior to COVID. COVID maybe has just accentuated some aspects of it. But this goes far beyond just the current pandemic situation… We need to address ways of trying to help the most vulnerable—those who are most profoundly affected through alcohol—and who our traditional sort of modalities of treatment and approach are not working for.*

(Stakeholder 9)

*I would quite like to see it being long term, yes. I don’t think it’s an alternative option. I don’t think necessarily that there is a service for everybody. I also don’t think fellowship is for everybody. Having another option that may help someone, that’s done in a different way. People need to find whatever is going to work for them.*

(Stakeholder 19)

## 4. Discussion

This mixed methods study investigated the potential of MAPs in The Salvation Army (TSA) homelessness services in Scotland in the context of COVID-19 and, most specifically, how this harm reduction intervention might prevent/reduce infection risk for people who experience homelessness and alcohol use disorders (AUDs). To be clear, no MAP was implemented in Scotland during COVID-19. We have described stakeholder, provider, and client views on the potential of MAPs as a response to COVID-19 and beyond. Presenting quantitative data from a small case note review, and qualitative data from 40 semi-structured interviews, we describe how people experiencing homelessness accessed alcohol, and wider health and social services, in the context of the pandemic. We articulate participant views on how MAPs might enable individuals to access alcohol more safely and thereby reduce acute health and social harms and potentially be protected from acquiring/transmitting COVID-19 by enabling them to receive alcohol in their residence. We also outline the fears and concerns that participants had in relation to operating MAPs in the context of COVID-19. Overall, MAPs were viewed as having potential in being able to address the current lack of services in Scotland for this structurally vulnerable group.

Our findings illustrate the impact of the COVID-19 pandemic in exacerbating the harms experienced by people experiencing alcohol dependence and homelessness in Scotland. While our findings highlight the opportunities presented by the pandemic to consider reducing or stopping alcohol consumption, many participants suggested that polysubstance use had increased, with people experiencing homelessness substituting other substances for alcohol. This was confirmed by the small case note review we undertook across three TSA homelessness settings. This review highlighted the complexity of people’s lives, including high rates of alcohol use, physical/mental health problems, polysubstance use, and the difficulty individuals had in complying with COVID-19 lockdown restrictions. The finding that 100% of individuals sampled in the case note review were also using illicit drugs is important to highlight, due to the additional risks for this group of harms such as overdose and drug-related deaths. This was also a feature of a Canadian study by Erikson et al. [32] that explored coping strategies utilised by 364 people with housing instability and AUD when alcohol became unavailable. Erikson et al. also highlighted that clients who accessed MAPs were more likely to access treatment, substitute illicit drugs, and experience fewer overdoses, compared with those not in MAPs. This is particularly relevant given the polysubstance use and high rate of overdoses in Scotland [57,58].

As part of the COVID-19 response, services were described as having made agile and innovative operational changes to meet need, including rapid accommodation provision, alternative support methods, and expanding the use of harm reduction approaches within accommodation services which are not abstinence-based. This connects with the literature gathered since the 1990s highlighting community-based harm reduction outreach as providing an essential first point of contact for people who use substances [59,60,61,62]. More generally, alcohol-specific harm reduction approaches for people experiencing homelessness are also increasing in a number of countries, including the US, in recognition of the need for such programmes, independently of COVID-19 [23,24,63]. These services could also be important providers of alcohol harm reduction education, such as the safer drinking guidelines developed during COVID-19 [40] and described by Grazioli et al. [64] on providing alcohol harm reduction within drop-in programmes.

In the context of COVID-19, MAPs have been proposed to be an important addition to services for people experiencing homelessness and AUD [19] to provide: health and well-being support; maintenance or reduction of alcohol intake in order to prevent withdrawal and prevent alcohol-related complications; elimination/reduction of non-beverage alcohol consumption; promotion of safer use through harm reduction education and self-management; reduction in risk/prevention of COVID-19 infection and transmission due to not having to leave accommodation to source alcohol; and support with isolation measures for suspected/confirmed COVID-19 cases. In our study, participants described a de-prioritisation of alcohol services in favour of services addressing other health conditions and illicit drug dependency, despite known risks for those with AUDs.

In British Columbia (BC), Canada, operational guidance for MAPs was released to address the harms of homelessness, AUD, and COVID-19 [30]. These programmes were significantly upscaled by health authorities, with operational guidance being approved by the BC Ministry of Health based on the identified need and request for such services by service providers alongside the loss of other services. The first MAPs in the US were established during the pandemic and provided support to 26 individuals across three settings in California, showing positive outcomes after two months [42]. The four emergency shelters set up in Lisbon in response to the COVID-19 pandemic also represent a relevant example of the potential of alcohol and drug harm reduction interventions integrated into rapid accommodation provided for people experiencing homelessness [65].

In terms of implications for policy and practice, our findings highlight the lack of harm reduction related to alcohol compared to illicit drugs. While drug-related deaths in Scotland are at an all-time high, our study has highlighted an important opportunity regarding the need to bring alcohol and drugs harm reduction together for those who are experiencing wider complex social problems such as homelessness. Our participants were also clear that MAPs should extend beyond being a response to COVID-19. While some clients found the new forms of online support helpful and accessible, others experienced these as limiting their support. Blended forms of in-person and online/phone supports might be merited going forward to build on the learning from the pandemic. In terms of research implications, it is important that research studies are conducted during times of severe challenge in health and social care services, despite the added burden that this can place on services. Community–university partnerships can be powerful in providing the infrastructure for such research at times of crisis because they capitalise on effective and established networks of trust and communication. Given there will be a MAP opening in Scotland in late 2021, we recommend rigorous evaluation of client outcomes to extend the evidence base of this particular model of alcohol harm reduction for people experiencing homelessness.

### Strengths and Limitations

In terms of strengths, the collaborative partnership between TSA and the research team facilitated data collection within a range of settings, enabling insight into both clients’ needs and wider views on MAPs. Involving a wide range of participants across different sectors (health, housing, homelessness, substance use) provided a detailed understanding of alcohol access and use during an unprecedented time. The curation of a series of paintings brought the study themes to life in a novel way. Further exploration of how original paintings might complement traditional qualitative methods is worth considering in future studies. The use of detailed field notes to capture researcher experiences and reflections enabled us to change the interview schedule to enhance clarify and capture more relevant data. Finally, we were able to collect data from a wide range of participants, including those who were deemed particularly vulnerable, at the height of the pandemic.

Several limitations were experienced during data collection. Firstly, as noted in the Methods section, it was difficult to identify participants for the case record review due to the mobility of this group: some who had been eligible at the start of the study had left the setting at the point of data collection. Secondly, the data captured in case notes was sometimes incomplete given data were not routinely captured by the settings which were in contingency mode due to COVID-19 (staff were prioritising contact/relationships). Relatedly, while some information was clearly provided in the case records, other information required calculation/interpretation (for alcohol units, mental health problems, alcohol-related cognitive impairments, other diagnoses, and medications). These data may therefore be an underestimation of people’s problems. Having a TSA manager collect the data did enable a more complete set, compared to having researchers undertake this task. It is important to highlight that severe staff shortages and high workloads, as a direct result of managing the pandemic, also meant that many staff were unable to participate in interviews or had little time to help the research team identify clients. Thirdly, service managers may have been biased regarding who to invite to take part in the study—both clients and staff members. Fourthly, because the study was only six months in duration (as per the funder’s ‘rapid response’ requirements), we were limited in the number of interviews we could conduct. This was particularly the case for client interviews: two were conducted in person but data collection had to revert to remote methods due to local pandemic restrictions. While we conducted phone interviews with an additional four clients, this approach was not suitable for all, especially those without access to technology/phones, or who felt uncomfortable participating for other reasons, which meant we interviewed far fewer clients than we had hoped. Additionally, due to the pragmatic sampling we had to conduct because of the COVID-19 pandemic, we were not able to capture a more diverse set of client views. Finally, it is also worth noting that the study was conducted within one organisation which limits transferability of our findings. However, we did include settings in two cities in different parts of the country that encompassed three very different service models. We also included a large number of stakeholders from other relevant organisations, and who were able to speak from a national perspective, to try to mitigate this.

## 5. Conclusions

This study aimed to understand the potential of MAPs in TSA settings in Scotland in the context of COVID-19 to reduce risks of infection and transmission of the virus for people experiencing homelessness and AUDs. Case record data (albeit from a small sample of people) highlight high rates of alcohol and drug use, and mental and physical health problems, with the majority reported to have broken government lockdown rules to secure alcohol. Qualitative analysis generated five subthemes under the dominant theme of ‘MAPs as a response to COVID-19′: changes to alcohol supply/use and polysubstance use; COVID-19-related changes to services; negative changes to services for people with alcohol problems; the potential of MAPs in the context of COVID-19; and fears and concerns about providing MAPs as part of a COVID-19 response. These data suggest that alcohol harm reduction services were perceived to be lacking in Scotland, both before and during the pandemic, with inequalities noted between policy and practice responses to those with alcohol problems and those with drug problems. The pandemic therefore offered an opportunity to more clearly discern the vulnerability of this group and associated gaps in provision. Alcohol harm reduction should be taken forward in Scotland to reduce generic risks/harms, as well as COVID-19-related risks/harms, for this vulnerable group. Implementing MAPs as a standard treatment/support approach could be a vital move towards improving the quality of life of people with AUDs who also experience homelessness and polysubstance use by offering options and choice.

## Figures and Tables

**Table 1:** Demographic information from case record review.

	*n* = 12
***Gender***	
Male	11
Female	1
***Age***	
30–39 years	3
40–49 years	6
50–59 years	1
60+ years	2
***Current housing status***	
Own home/secured tenancy	8
Hostel	4
***Current service setting***	
Housing First	7
Residential	4
Drop-in	1
***Source of income***	
Benefits	12
***Physical health problems***	
Physical health problems reported	11
Mobility/joint problems	4
Respiratory problems	3
Underweight	1
Liver damage	1
Epilepsy	1
Other	1
None	1
***Ambulance call-outs***	
Ambulance call-outs	8
Never had an ambulance call-out	2
No data	2
***Mental health problems***	
Mental health problems reported	12
Depression and/or anxiety	11
PTSD/trauma	2
ARBD	1
Psychosis/schizophrenia	1
***Number of mental health problems reported***	
More than one mental health problem reported	10
One mental health problem reported	2

**Table 2:** Alcohol and drug use information from participant case records.

	*n* = 12
***Approximate units per day***	
10–19 units	1
20–29 units	8
30–39 units	2
>40	1
***Days drinking per month***	
10–14	1
15–19	1
20–24	2
25–29	2
Everyday	6
***Type of alcohol***	
Beer/cider	5
Wine	1
Spirits	1
More than one type	5
***Means of purchasing alcohol***	
Purchases own alcohol	7
Partner or friend buys alcohol	2
Pools resources to buy alcohol	3
***Number of years drinking***	
10–20 years	1
20–30 years	6
>30 years	5
***NBA use***	
Does not drink NBA	12
***AUDIT alcohol dependence score***	
Number of participants with AUDIT score	11
Score range	13–36
Mean AUDIT score *	30
Median AUDIT score *	33
***Seizures***	
History of seizures or current seizures	7
No experience of seizures	5
***Withdrawal symptoms***	
Daily withdrawal symptoms	12
Previous alcohol treatment	
Experience of treatment	4
No experience of treatment	6
No data	2
***Previous alcohol detoxification episodes***	
Experience of detoxification	6
No experience of detoxification	3
No data	3
***Alcohol-related hospital admissions***	
Experience of hospital	8
No hospital admissions	2
No data	2
***Alcohol-related ambulance call-outs***	
Experience of ambulance call-out	8
No experience	2
No data	2
***Alcohol-related cognitive impairments***	
Cognitive impairments reported	8
No cognitive impairments reported	4
***Drug use***	
Current drug use reported	12
Cannabis	3
Cocaine/crack cocaine	1
Benzodiazepines	1
More than one substance	7

* AUDIT scores range from 0–40, with scores of 8–15 indicative of hazardous drinking; 16–19 of harmful drinking; and 20–40 of alcohol dependence [53].

**Table 3:** COVID-19-related data.

	*n* = 12
***Tested for COVID-19***	
Not tested	11
Tested	1
***COVID-19 symptoms***	
No symptoms displayed	11
Symptoms displayed	1
***Shielding***	
Not shielding	11
Shielding	1
***Lockdown rules***	
Broke lockdown rules	9
Kept lockdown rules	3

**Table 4:** Interview participant characteristics.

Participants and Organisations	
***External stakeholders (n = 19)***	
Health	*n* = 11
Statutory	*n* = 4
Other	*n* = 4
***TSA service managers (n = 8)***	
National role	*n* = 3
Frontline service manager	*n* = 5
***TSA frontline staff (n = 7)***	
Setting 1	*n* = 4
Setting 2	*n* = 2
Setting 3	*n* = 1
***Potential beneficiaries/clients (n = 6)***	
Setting 1	*n* = 1
Setting 2	*n* = 2
Setting 3	*n* = 3

## Data Availability

The datasets generated and/or analysed during the study are not publicly available. Individual privacy could be compromised if the dataset is shared due to the small sample involved.

## Supplementary Materials

The following are available online at www.mdpi.com/article/10.3390/ijerph182312523/s1, Supplementary File S1: Interview schedules for all groups.

## Abbreviations

ARBD	Alcohol Related Brain Damage
AUD	Alcohol Use Disorder
AUDIT	Alcohol Use Disorders Identification Test
GUEP	Stirling’s General University Ethics Panel
MAPs	Managed Alcohol Programs
NBA	Non-Beverage Alcohol
ONS	Office for National Statistics
PPE	Personal Protective Equipment
PTSD	Post Traumatic Stress Disorder
SG	Strategy Group
SHAAP	Scottish Health Action on Alcohol Problems
TSA	The Salvation Army
WHO	World Health Organization
