# Supplementary material for: Exploring the Potential of Implementing Managed Alcohol Programmes to Reduce Risk of COVID-19 Infection and Transmission, and Wider Harms, for People Experiencing Alcohol Dependency and Homelessness in Scotland
Source: Int J Environ Res Public Health. 2021 Nov 28;18(23):12523. doi: 10.3390/ijerph182312523 (PMC8657286; doi:10.3390/ijerph182312523)
Supplement: Supplementary file 1 [file ijerph-18-12523-s001.zip › ijerph-1419523-Supplementray.pdf]

Supplementary File S1: Interview schedules for all groups

Client/potential beneficiary participants

How long have you lived here/been using this service?

This is a study about how to provide good support to individuals with problem alcohol use. Do you consider yourself to have problems with alcohol?
Probes: Would you mind telling me more about any problems you have in different areas of your life? Are any of these created by drinking too much, through challenges accessing alcohol or made worse by drinking?

If you are comfortable doing so, please can you tell me a bit more about your drinking? *Probes: How much? How often? What times of day? Do you ever drink any non-beverage alcohol (like mouthwash, methylated spirits, rubbing alcohol, hand sanitiser)? Do you drink on your own or with others? In the service or outside of the service you are currently in, or both?*

Have you ever tried to access alcohol treatment (including rehabilitation/ detoxification)? If yes, how many times?
Probes: How did you find that treatment? Please tell me about the things that worked or did not work for you if you are comfortable doing so (with probes around how many times they have experienced detox and how soon physical dependence occurs after drinking restarts).

How have you found accessing alcohol during the Covid-19 pandemic?
Probes: Where have you got it from (purchased or from others)? Who has got it for you? Have you had enough alcohol? Have you used other substances when you couldn't access alcohol? Are you drinking the same alcohol (type, brand, amount) as before? Has anything else about your alcohol use changed?

What changes have you seen in this service during the pandemic? What about in other services?
Probes: What have you thought about these changes? Would you like them to continue? Why/why not?

What help/support/treatment options would you have liked to have seen for your alcohol use during this pandemic?
Probes: Why? How would this have helped?

If a MAP was delivered in this service, would you use it?
Probes: Do you think it might help you? Do you think it would affect your risk of getting covid-19? In what way?

If a MAP was to be delivered in this service, what would it need to include?
Probes: Would you want it to be residential/drop in? Provide different activities/services? Provide different options for alcohol use? Anything else?

Is there anything else you'd need to feel supported/comfortable if a MAP was introduced here?
Probes: What else might be beneficial in helping you with alcohol use during the pandemic?

What things might prevent the MAP from being delivered here?
Probes: Who might not want the MAP to be delivered here? Do you think these barriers could be removed?

What lessons can be learned from your experiences of alcohol use/homelessness during the pandemic?

Is there anything else you'd like to add?

Staff/service managers

What is your role/day to day job?

What are your experiences of working with/supporting people with problem alcohol use?

What are your experiences of supporting people with problem alcohol use during the Covid-19 pandemic?

What changes have been made in the service to deal with the pandemic?
Probes: How were these changes received by staff/service users?

What is your understanding of Managed Alcohol Programmes (MAPs)?
Probes: How did you first hear about MAPs? What was your first impressions of them? Have you received any information/training on MAPs from TSA or externally?

What are your thoughts about the potential of MAPs during the pandemic?
Probes: Would they be beneficial? Why/why not?

How do you think clients would respond to the approach?
Probes: Why would they be positive/negative about MAPs?

Do you think a MAP would affect participants' risk of getting covid-19? In what way?
Probes: What other things might reduce/increase their risk?

If a MAP was to be implemented in this service, what would facilitate and support this?
Probes: What support/help do you think you would need to work in the MAP? (e.g. training, reflective practice, emotional support, practical support)

What, if anything, would act as a barrier to implementation?
Probes: Why? What could be done to overcome this?

How best do you feel that you could be supported to deliver and contribute effectively a MAP within your service?

What lessons can be learned from the way in which people's alcohol use has been managed during the pandemic?

Is there anything else you'd like to add?

Wider stakeholders

What is your role/day to day job?

Thinking about problem alcohol use, what are the typical treatment pathways for people who are homeless?
Probes: What are your experiences of working with/supporting people with problem alcohol use who are homeless?

What are your experiences of supporting people with problem alcohol use **during** the Covid-19 pandemic?
Probes: If no direct experience, are you aware of other people's experiences of working with people with problem alcohol use during Covid-19?

What changes have you seen across services to deal with the pandemic?

Probes: Have you seen an increase in risk for people with problem alcohol use (e.g. in terms of alcohol availability)?
How have services responded to these risks?
How have changes to services been received by staff/service users/others?

What is your understanding of Managed Alcohol Programmes (MAPs)?
Probes: How did you first hear about MAPs?
What was your first impressions of them?
Have you received any information/training on MAPs?

What are your thoughts on the potential for MAPs during the pandemic?
Probes: Would they be beneficial? Why/why not?

Do you think a MAP would affect participants' risk of getting covid-19?
Probes: In what way?

How do you think clients would respond to the approach?
Probes: Why would they be positive/negative about MAPs?

If a MAP was to be implemented in third sector homelessness services, what would facilitate and support this?
Probes: What would need to be put in place to ensure it was delivered successfully? (Staffing, training, location, residential vs. day services, licensing, buy-in, funding etc)
Should clients be involved in informing how MAPs are implemented? How could this be done best?

What, if anything, would act as a barrier to implementation?
Probes: Why? What could be done to overcome this?

Do you think these MAPs should be a Covid-19 response or more long-term?

In summary, what lessons can be learned from the way in which people's alcohol use has been managed during the pandemic?

Is there anything else you'd like to add?
